# Associations of Serum Concentrations of Organochlorine Pesticides with Breast Cancer and Prostate Cancer in U.S. Adults

**DOI:** 10.1289/ehp.0900919

**Published:** 2009-09-03

**Authors:** Xiaohui Xu, Amy B. Dailey, Evelyn O. Talbott, Vito A. Ilacqua, Greg Kearney, Nabih R. Asal

**Affiliations:** 1 Department of Epidemiology and Biostatistics, College of Public Health and Health Professions, University of Florida, Gainesville, Florida, USA;; 2 Department of Epidemiology, Graduate School of Public Health, University of Pittsburgh, Pittsburgh, Pennsylvania, USA;; 3 Environmental Health Program, College of Public Health and Health Professions, University of Florida, Gainesville, Florida, USA;; 4 Florida Department of Health, Division of Environmental Health, Tallahassee, Florida, USA

**Keywords:** cancer, endocrine disruptors, organochlorine pesticides, pesticide, prostate cancer

## Abstract

**Background:**

Organochlorine (OC) pesticides are a group of environmental endocrine disruptors that may be associated with an increased risk for hormone-related cancers including cancers of the breast and prostate. However, epidemiologic evidence is limited and inconsistent.

**Objectives and methods:**

We used 1999–2004 National Health and Nutrition Examination Survey data to examine associations between serum concentrations of OC pesticides and prostate and breast cancers.

**Results:**

After adjustment for other covariates, serum concentrations of β-hexachlorocyclohexane (HCH) (*p* for trend = 0.02), *trans*-nonachlor (*p* for trend = 0.002), and dieldrin (*p* for trend = 0.04) were significantly associated with the risk of prevalent prostate cancer. Adjusted odds ratios for the second and third tertiles of detectable values were 1.46 [95% confidence interval (CI), 0.52–4.13] and 3.36 (95% CI, 1.24–9.10) for β -HCH; 5.84 (95% CI, 1.06–32.2) and 14.1 (95% CI, 2.55–77.9) for *trans*-nonachlor; and 1.06 (95% CI, 0.30–3.73) and 2.74 (95% CI, 1.01–7.49) for dieldrin compared with concentrations in the lowest tertile or below the limit of detection. However, there was no positive association between serum concentrations of OC pesticides and breast cancer prevalence.

**Conclusion:**

Although further study is necessary to confirm these findings, these results suggest that OC pesticide exposures may have a significant effect on cancer risk. Efforts to reduce worldwide OC use are warranted.

Organochlorine (OC) pesticides, a class of hydrocarbon compounds characterized by their cyclic structure, number and position of chlorine atoms, and low volatility, were widely used in agriculture and pest control after they were introduced in the 1940s. They include hexachlorocyclohexane (HCH) isomers, DDT and its analogs, and cyclodienes. Because of their nature of persistence in the environment, bioaccumulation in the food chain, and possible health effects, the U.S. Environmental Protection Agency restricted and banned the use of most of OC pesticides during the 1970s and 1980s. Although OC pesticides are rarely used in the United States today, measurable amounts of OC pesticides or their metabolites can still be found in human tissues in the United States. Moreover, OC pesticides continue to be heavily used in some developing countries, primarily for mosquito and malaria control (Turusov et al. 2002). Thus, the health effects of OC exposure remain an important global public health concern.

Evidence from experimental assays suggests that a number of OC pesticides demonstrate weak estrogenic or antiestrogenic effects (Soto et al. 1995). These chemicals interfere with the function of the endocrine system by mimicking a hormone, blocking the effects of normal, endogenous hormones, or by altering or modifying the synthesis, metabolism, or transport of hormones (Soto et al. 1995). It is believed that these compounds may act as a tumor promoter through hormone-mediated effects (Hansen and Matsumura 2001). Some epidemiologic studies have linked OC pesticides with several hormone-related cancers including breast cancer (Calle et al. 2002), prostate cancer (Mills and Yang 2003), endometrial cancer (Weiderpass et al. 2000), and testicular cancer (Biggs et al. 2008). However, findings from epidemiologic studies have not been consistent, particularly for breast and prostate cancer.

The primary source of exposure to OC pesticides in the general population is believed to be through diet via consumption of milk, fish, and meat (Toppari et al. 1996). Additional exposure pathways include dust, air, and soil. Measurement of OC pesticide exposure is a complex and challenging process because of the multiple pathways of exposure. Biomonitoring of exposure is a useful tool for assessing exposure to OC pesticides. Specifically, measurement of OC pesticides, their metabolites, or reaction product in biological media such as blood or urine is an effective way to determine the extent of chronic exposure to these chemicals (Barr 2008). In the National Health and Nutrition Examination Survey (NHANES), a subsample of one-third of participants ≥ 12 years of age was selected and was measured for the concentrations of OC pesticides or their metabolites in blood in each biannual data cycle. In addition, information on personal medical conditions including physician- diagnosed site-specific cancers was collected using a questionnaire. In this study, we used the 1999–2004 NHANES data to evaluate the relationships between lipid-adjusted serum concentrations of OC pesticides and breast cancer as well as prostate cancer.

## Materials and Methods

### Study population

Data from the NHANES survey cycles conducted in 1999–2000, 2001–2002, and 2003–2004 were obtained online (CDC 2009d). During these three data collection cycles, blood samples from the one-third of the participants ≥ 12 years of age were collected and measured for serum concentrations of several OC pesticides or their metabolites. For each NHANES data cycle, this random subsample is representative of the noninstitutionalized U.S. civilian population. Participants < 20 years of age were not asked for information about chronic medical conditions including a diagnosis of site-specific cancer. Consequently, this analysis included only participants who were both tested for blood OC pesticides and asked for information about personal medical conditions. A total of 1,475 participants for the 1999–2000 cycle, 1,693 participants for the 2001–2002 cycle, and 1,585 participants for the 2003–2004 cycle were eligible for analysis of the associations of breast cancer as well as prostate cancer and OC pesticide exposure. Furthermore, individuals with other types of cancer were excluded from this analysis. Finally, the final sample size varied because of missing values of the dependent variable, the exposures, and the selected covariates. This study was conducted in conformity with all applicable requirements of the United States and/or international regulations of research on human subjects. The research protocol was reviewed and approved by the University of Florida Institutional Review Board.

### OC pesticide measurements

OC pesticides were measured in serum by high-resolution gas chromatography/isotope-dilution high-resolution mass spectrometry. Blood was collected in red-top Vacutainers, which were placed upright in a rack to allow blood to clot at room temperature for 20–30 min. Serum was collected and frozen within 1.5 hr after the specimen was collected. Each analytical run consisted of nine unknown specimens, one method blank, and two quality control samples. The limit of detection (LOD) for each analyte was calculated correcting for sample weight and recovery. In addition, the total lipid content of each specimen was estimated from its total cholesterol and triglycerides values using a summation method. Analytical results for OC pesticides are reported on a lipid-adjusted basis (nanograms per gram or parts per billion). The lipid-adjusted concentration of an analyte is given by


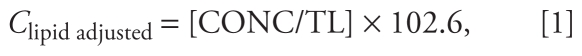


where CONC is the concentration of an analyte in a sample as weight per gram of sample, and TL (total lipid) = (2.27 × total cholesterol mg/dL + triglycerides + 62.3). The details of the laboratory protocol are available online (CDC 2006, 2009a, 2009b). Ten serum OC pesticides or their metabolites were measured in these three NHANES data cycles (1999–2000–2001–2002, and 2003–2004). These 10 biomarkers included hexachlorobenzene, β-HCH, G-HCH, *p,p*′-DDE (dichlorodiphenyldichloroethylene), *p,p*′-DDT (dichlorodiphenyltrichloroethane), *o,p*′-DDT, oxychlordane, *trans*-nonachlor, heptachlor epoxide, and mirex. The other three OC pesticides including aldrin, dieldrin, and endrin were measured only in NHANES 2001–2002 and NHANES 2003–2004 data cycles. To minimize the nondifferential measurement error among those below the LOD, we selected the OC pesticides for which at least 50% of study subjects had serum concentrations above the LOD. In our analyses, the selected chemicals include β-HCH, *p,p*′-DDE, oxychlordane, *trans*-nonachlor, heptachlor epoxide, and dieldrin. Table 1 presents the information regarding the number of missing values and the value range for each selected OC pesticide in each data cycle.

### Covariates

Covariates related to the development of hormone-related cancers were selected and controlled for while evaluating the associations between exposure to OC pesticides and cancers. Participants’ demographic information such as age (< 65 years vs. ≥ 65 years of age), sex, race/ethnicity (non-Hispanic white, non-Hispanic black, and others that included Mexican Americans, other Hispanics, other races/multiracial individuals), education (> high school, high school, < high school), marital status (yes/no), poverty/income ratio (< 1.0, 1.0–2.0, or > 2.0), and lifestyle factors such as cigarette smoking (yes/no) and alcohol drinking (yes/no) were extracted from the personal interview questionnaire. Other covariates such as body mass index (BMI; < 25.0, 25–29, or ≥ 30) and time since cancer diagnosis, which was estimated by subtracting age at cancer diagnosis from age at survey, were also selected. Considering potential temporal trend of OC pesticides in the U.S. population, data cycle (1999–2000–2001–2002–2003–2004) was also included as a covariate in the analysis.

For breast cancer, additional covariates of reproductive health such as age at menarche (≤ 12, 13–15, and ≥ 16 years), menopausal status (yes vs. no), parity (nulliparous, 1, 2, 3, and ≥ 3 births), age at first full-term birth or nulliparity (< 25 years, 25–29 years, ≥ 30 years, or nulliparity), ever breast-fed child (yes vs. no), oral contraceptive use (current, ever, never), and ever used hormone replacement therapy (yes vs. no) were also examined.

### Cancer outcomes

Medical conditions were collected using the NHANES questionnaire. The following questions were used to ascertain individual cancer status: “[Have you/Has SP] ever been told by a doctor or other health professional that [you/s/he] had cancer or a malignancy of any kind?” and if yes, “What kind of cancer is it?” According to the questions, the individual was asked to report physician-diagnosed cancers. There was no further validation of the cancer diagnosis in these data. In this analysis, individuals who reported breast cancer or prostate cancer were selected as the case group. Other types of cancers were excluded from this analysis.

### Data analyses

All statistical analyses were performed using SAS 9.1 software (SAS Institute Inc., Cary, NC) survey procedures, which account for the complex sampling design used in NHANES. The sample weights, stratification, and clustering design variables were incorporated into all SAS survey procedures to ensure the correct estimation of sampling error. A 6-year subsample weight was calculated for the combined 1999–2004 data by following the NHANES analytic and reporting guidelines and assigning one-third of the 2-year OC pesticides subsample weight (WTSB2YR) for 2003–2004 if a participant was sampled in 2003–2004 and merging it with two-thirds of the 4-year 1999–2002 OC pesticides subsample weight (WTSPO4YR) for those sampled in 1999–2002 (CDC 2009c). We used this calculated weight to analyze the merged 6-year data of NHANES 1999–2004 data. Because dieldrin was measured only in the NHANES 2001–2004 data cycles, a 4-year subsample weight was calculated for the combined 2001–2004 data by assigning half of the 2-year OC pesticides subsample weight (WTSB2YR) for 2003–2004 if a participant was sampled in 2003–2004 and merging it with half of the 2-year 2001–2002 OC pesticides subsample weight (WTSPO2YR) for those sampled in 2001–2002.

Descriptive statistics such as two-sided Student *t*-tests and Wald chi-square analyses were also performed, where appropriate. Logistic regression models were used to evaluate the associations between exposure to OC pesticides and hormone-related cancers, breast cancer or prostate cancer. For each OC pesticide, the reference group is defined as those participants whose serum concentrations are less than the LOD, and subjects with detectable values were categorized by cutoff points of 33rd and 67th percentile values, which were calculated only among those with values above the LOD in the whole study population including both cases and noncases. If there were no cancer cases or a small number of cancer cases (≤ 5 cases) in the reference group, subjects within the first tertile of detectable values were also included in the reference. We used the Akaike Information Criterion (AIC) to determine the best-fitting model. If two models were nested models, the more parsimonious model was selected if the χ^2^ difference was not significant based on AIC. Otherwise, the smaller AIC was used as indicator of the better fit. Moreover, we proposed several models adjusting for different sets of covariates to examine the associations between OC pesticides and cancer risk to determine how robust the results were. Specifically, age-adjusted associations between exposure to individual chemicals and breast cancer as well as prostate cancer were calculated. Furthermore, multivariate logistic regression was used to examine these associations, with adjustment for known risk factors and potential confounders. Odds ratios (ORs) and 95% confidence intervals (CIs) were reported.

## Results

The sample of 4,237 participants included 4,109 individuals without cancer and 128 cancer cases including 63 breast cancer cases and 65 prostate cancer cases. Relationships between all 21 pairwise combinations of six OC pesticides as well as time since cancer diagnosis are presented in Table 2, with results of the Spearman rank correlation tests. The results indicated that significant correlations exist between all OC pesticides pairwise combinations. The Spearman rank correlation coefficients ranged from 0.38 to 0.91. Oxychlordane was highly correlated with *trans*-nonachlor (0.91), heptachlor epoxide (0.65) and β-HCH (0.61). β-HCH was also highly correlated with *p,p*′-DDE (0.73). Time since cancer diagnosis was not correlated with any of the OC pesticides, with correlation coefficients ranging from –0.14 to 0.20.

Table 3 describes the relationships between selected characteristics and OC pesticides among the study population. Age was significantly associated with presence of OC pesticides. Individuals in the older age group had significantly higher concentrations of OC pesticides than those in the younger group. The percentages in the third tertiles of OC pesticides for younger (< 65 years of age) versus older (≥ 65 years) are 10.4% versus 51.4% for β-HCH; 16.0% versus 48.7% for *p,p*′-DDE; 12.4% versus 68.1% for oxychlordane; 15.7% versus 65.6% for *trans*-nonachlor; 12.5% versus 39.2% for heptachlor epoxide; and 18.4% versus 49.4% for dieldrin, respectively. In addition, lower education, higher BMI, and being a former smoker were significantly associated with higher exposure to OC pesticides. The associations between OC pesticides and other covariates including race/ethnicity, family income, marital status, and alcohol consumption varied across specific chemicals.

Table 4 presents the distribution of selected characteristics of the 1999–2004 NHANES adult participants by breast and prostate cancer status, respectively. The adults who were diagnosed with breast cancer were more likely than those without cancer to be in the older age group (57.5% vs. 15.6%, *p* < 0.001), white (92.6% vs. 69.1%, *p* < 0.001), and of higher income level (68.0% vs. 61.0%, *p* = 0.02). However, there was no significant difference in alcohol use, smoking, BMI, and marital status between participants diagnosed with breast cancer and those without cancer. In addition, prostate cancer cases were also more likely than those without cancer to be older age (85.7% vs. 11.7%), white (83.2% vs. 72.1%), and of higher income level (97.1% vs. 88.6%). Moreover, prostate cancer cases were more likely than those without cancer to have a lower education level (30.2% vs. 20.6%) and less alcohol use (31.6% vs. 16.4%).

Table 5 shows associations between the six selected OC pesticides and the prevalence of prostate cancer. We present the results from both age-adjusted and multiple variable adjusted models. From the age-adjusted model, we found that β-HCH, oxychlordane, *trans*-nonachlor, and dieldrin showed significant trends with the prevalence of prostate cancer (all *p* for trend < 0.05). After adjustment for age (< 65 years of age vs. ≥ 65 years), race/ethnicity, BMI, education, smoking, NHANES data cycle, and marital status, the trends for prevalence of prostate cancer remained significant for three of six OC pesticides including β-HCH, *trans*-nonachlor, and dieldrin. Compared with the reference group, adjusted ORs for the second and the third tertiles (respectively) were 1.46 (95% CI, 0.52–4.13) and 3.36 (95% CI, 1.24–9.10) for β-HCH (*p* for trend = 0.02); 5.84 (95% CI, 1.06–32.2) and 14.1 (95% CI, 2.55–77.9) for *trans*-nonachlor; and 1.06 (95% CI, 0.30–3.73) and 2.74 (95% CI, 1.01–7.49) for dieldrin.

We also examined the associations between prevalence of breast cancer with these six chemicals. However, none of the OC pesticides were found to be significantly associated with prevalence of breast cancer in age-adjusted models or multiple variable adjusted models, which included the covariates of age (< 65 years of age vs. ≥ 65 years), race/ethnicity, BMI, education, smoking, NHANES data cycle, and marital status. The results are presented in Table 6. We also considered other covariates including alcohol use, age at menarche, menopausal status, parity, age at first full-term birth or nulliparity, breast-feeding, oral contraceptive use, and hormone replacement therapy use in the adjusted models. However, the results did not appreciably change with further adjustment for these variables other than increasing the uncertainty of the estimation. The data are not presented.

## Discussion

Several studies have suggested that OC pesticides may modulate steroid sex hormones such as estrogen or testosterone as agonists or antagonists or as mixed effects (Sonnenschein and Soto 1998). Because some cancers (including those of the breast and prostate) are hormone-dependent, research has been focused on the potential risk associated with OC pesticides exposures (Ejaz et al. 2004). In this study, we examined the associations between OC pesticides and prostate cancer as well as breast cancer. This cross-sectional study demonstrated that background exposure to OC pesticides was positively associated with the prevalence of prostate cancer in the U.S. general population. The associations were consistently in the same direction, although the details of associations were substantially different depending on the specific subclass of OC pesticides.

Other epidemiologic studies have also suggested an association between prostate cancer and OC pesticides. Our findings are in general agreement with, but stronger than, those of previous epidemiologic studies. Several occupational cohort studies and case–control studies have found OC pesticides to be associated with an increased risk of prostate cancer (Dich and Wiklund 1998; Fleming et al. 1999; Ritchie et al. 2003; Settimi et al. 2003). However, some studies have not observed similar associations (Mozzachio et al. 2008; Wiklund et al. 1989). Thus, although our study adds to the growing evidence that OC exposure is associated with cancer prevalence, additional studies are necessary.

Although we observed a significant association between OC pesticides and prostate cancer prevalence, we did not observe an association with breast cancer prevalence. Although a few studies support the association between breast cancer and OC pesticide (Charlier et al. 2003; Wolff et al. 1993), the vast majority of epidemiologic studies do not (Ward et al. 2000; Wolff et al. 2000; Zheng et al. 2000). Overall, there is not overwhelming evidence that suggests that exposure to OC pesticide is associated with breast cancer (Calle et al. 2002).

Our study had some important design features that strengthen confidence in its results: First, we found considerable differences of the proportions of at or above LOD in the different data cycles for those pesticides that have a low proportion of values above the LOD (i.e., below 50%) in Table 1. One possible explanation could be that these pesticides with a very low proportion of values above the LOD are more sensitive to measurement errors introduced during sample collection, storage, preparation, and testing. Moreover, for the same OC pesticide, the detection limit varies by individual. The larger the serum volume, the lower the detection limits are. Therefore, the different study population sample and the volume of blood sample collected from each participant might also contribute to the variation of the percentage above the LOD in different years. To minimize nondifferential measurement error, we selected those OC pesticides that had at least 50% of study subjects with serum concentration above the LOD. Second, serum levels of individual OC pesticides represent surrogate measurements of long-term exposure. This methodology is regarded as the most effective way of assessing exposure to these chemicals, (i.e., lipophilic, resistant to metabolism, and stored in adipose tissue) (Barr 2008). Most previous studies have focused on highly selected populations such as a specific occupational group. The exposure pattern and dose of OC pesticides from occupational sites may be different from background exposures experienced by the general population. It has been shown that OC measurements in the blood of occupational populations may be 1–3 orders of magnitude higher than blood levels measured in the general population (Liem et al. 2000). However, few studies of OC pesticides have been carried out in the general population, even in a cross-sectional design, because of the high cost of collecting blood samples and the wide variation of OC pesticides or metabolites. Therefore, the NHANES data provided a unique chance to investigate the possible associations between serum concentrations of individual OC pesticides and hormone-related cancers in a representative sample of the general population. Finally, the NHANES data provided information regarding a variety of potentially confounding factors such as demographic characteristics, socioeconomic status, lifestyle factors, and physical measurements. Thus, we were able to control for the confounding effects of these factors in our analyses.

Nevertheless, this study also has several limitations. The findings from this study should be interpreted with caution because of the nature of a cross-sectional study design, despite both the magnitude of association and the dose–response relationship. OC pesticides display high resistance to degradation. Although the persistence of DDE, β-HCH, chlordane, heptachlor epoxide, and dieldrin in the environment varies by climate, these chemicals may persist in soil for a period ranging from a few years to decades. For example, the half-life of DDE in soil may be more than 20 years (Agency for Toxic Substances and Disease Registry 2002). Biological half-lives of several years have been reported in humans for these chemicals (Morgan and Roan 1971). Thus, it is possible that these chemicals could be stored in human body fat for a long time. Furthermore, because these chemicals are stored in fat, it is believed that adipose tissue loss could result in increased organ and blood concentrations of these compounds (Hue et al. 2006). Several studies suggested that weight loss may be associated with an increase in serum concentrations of OCs because of bioconcentration (Chevrier et al. 2000; Imbeault et al. 2002). Thus, if changes in body weight occur as a consequence of the development of cancers, the cross-sectional findings in this study may reflect reverse causality. We examined the correlations between time since cancer diagnosis and serum concentrations of OC pesticides to see if any clear pattern emerged that would indicate that cancer-related weight loss over time may be associated with OC concentrations. The results indicated that there was no association. In addition, body fat is an important risk factor for breast cancer and prostate cancer (Cleary and Grossmann 2009; Hsing et al. 2007). Although we adjusted for BMI in our analyses, BMI as a measure of body fat has certain limitations. It does not account for skeletal size, muscle mass, amount of body water, and the effect of sex (Charbonneau-Roberts et al. 2005). Therefore, additional measures of body fat, such as waist-to-hip ratio, should be considered in future studies. In addition, all cancers in this study were prevalent cancer cases, which could introduce survival bias. Although survival bias may be a concern, there is some evidence that OC pesticides also have adverse effects on cancer survival (Hoyer et al. 2000, 2001). Under such circumstances, we would expect survival bias to bias our estimates toward the null. Additionally, previous studies have reported that Mexican Americans have higher OC pesticide levels than the rest of the population (Bradman et al. 2007). Because of the limited number of cancer cases in this analysis, particularly among Hispanics, it was not possible to specifically examine the relationship between OC pesticides and prostate and breast cancers within this subpopulation. Moreover, measurement errors in exposures, covariates, and health outcomes are also of concern. Measurement errors in blood OC pesticides could stem from within-person variation of serum concentrations of OC pesticides and potential errors during sampling, storage, analysis of biological specimens, and data processing. Additionally, information regarding both health outcomes and covariates largely relied on self-reported data. Reliance on self-reported data in the measurement of both the dependent variables and some covariates raised concerns about the validity of causal conclusions for a range of reasons, including systematic response distortions and method variance (Mackay et al. 2007). Furthermore, the observed associations are potentially confounded by those unmeasured factors such as family history, previous dietary information, and previous physical activities. Finally, although the cohort as a whole provides adequate power to examine our primary research questions, the sample sizes within cancer case groups are relatively small. Consequently, there are relatively wide CIs of the estimations after accounting for multiple risk factors.

## Conclusion

Our analysis of the 1999–2004 NHANES data suggests that serum concentrations of OC pesticides were positively associated with hormone-related cancer as a group and with prostate cancer specifically in this sample of the U.S. population. Future studies should consider maximizing the case sample, improving the data quality by minimizing the proportion of missing data, increasing the information accuracy, and collecting additional information such as genetic factors, which may increase the value of the NHANES data sets to better address these questions. Because these findings are based on survey data with limited control over the measurement of variables, we suggest that examination of these relationships warrants further study. Importantly, prospective follow-up of cancer cases should be considered to address some of the methodologic concerns noted in this article, such as cancer-related weight loss and OC concentrations, in addition to exploring the effects of OC concentrations on survival. Therefore, prospective studies of the relation between background pesticide exposure and validated cancer diagnoses should be a priority in future studies. Despite the controversial and complex issues involved with continued OC use, efforts to improve cancer surveillance, early detection, and access to appropriate treatments should be considered in countries where OC pesticide use remains common.

## Figures and Tables

**Table 1:** Lipid-adjusted serum concentration of OC pesticides in 1999–2004 NHANES adult participants ≥20 years of age.

	1999–2000 cycle (*n* = 1,475)			2001–2002 cycle (*n* = 1,693)			2003–2004 cycle (*n* = 1,585)		
Chemical	No. (missing)	Percent ≥LOD	Value range (ng/g)	No. (missing)	Percent ≥LOD	Value range (ng/g)	No. (missing)	Percent ≥LOD	Value range (ng/g)
Hexachlorobenzene	1,111 (364)	26	12.4–381.0	1,530 (163)	7.52	3.3–241.0	1,373 (212)	0.07	1.55–174.0
β-HCH	1,240 (235)	83.23	1.46–766.36	1,533 (160)	72.74	1.41–3500.0	1,370 (215)	79.56	0.90–2850.0
λ-HCH	1,139 (336)	3.95	1.6–127.0	1,522 (171)	1.12	0.4–73.3	1,367 (218)	0.66	0.35–304.0
*p,p*′-DDE	1,278 (197)	99.92	6.58–27900.0	1,540 (153)	99.94	3.75–24200.0	1,368 (217)	99.85	1.56–22900.0
*p,p*′-DDT	1,002 (473)	43.01	3.25–3450.0	1,549 (144)	38.67	2.33–2280.0	1,370 (215)	81.75	0.90–676.0
*o,p*′-DDT	1,002 (473)	1.1	2.2–116.0	1,523 (170)	2.1	0.7–194.0	1,358 (227)	7.51	0.49–223.0
Oxychlordane	998 (477)	82.36	2.3–218.0	1,497 (196)	81.96	1.7–289.0	1,383 (202)	87.85	1.06–159.0
*trans*-Nonachlor	1,269 (206)	91.57	2.3–331.0	1,528 (165)	89.20	1.7–834.0	1,366 (219)	96.19	1.34–355.0
Heptachlor epoxide	951 (524)	52.58	2.3–912.0	1,518 (175)	60.21	1.4– 181.0	1,371 (214)	66.67	1.10–154.0
Mirex	1,194 (281)	13.32	1.6–22.0	1,529 (164)	33.75	1.1–2960.0	1,359 (226)	49.45	0.49–166.0
Aldrin	–	–	–	1,519 (174)	0.26	0.4–8.5	1,358 (227)	0.15	0.35–58.1
Dieldrin	–	–	–	1,443 (250)	64.73	1.9– 670.0	1,365 (220)	90.70	1.20–448.0
Endrin	–	–	–	1,457 (236)	0.14	0.4–11.7	1,286 (299)	0.16	0.35–6.10

**Table 2:** Spearman correlations (no. of samples) between OC pesticides/metabolites as well as time since cancer diagnosis in 1999–2004 NHANES adult participants ≥20 years of age.

	β-HCH	*p,p*′-DDE	Oxychlordane	*trans*-Nonachlor	Heptachlor epoxide	Dieldrin	Time since cancer diagnosis^a^
β-HCH	1.0	0.73* (3,841)	0.63* (3,569)	0.61* (3,822)	0.58* (3,549)	0.43* (2,594)	0.09 (107)
*p,p*′-DDE	—	1.0	0.53* (3,587)	0.56* (3,867)	0.44* (3,561)	0.38* (2,605)	− 0.02 (106)
Oxychlordane	—	—	1.0	0.91* (3,578)	0.65* (3,540)	0.53* (2,569)	0.20 (97)
*trans*-Nonachlor	—	—	—	1.0	0.64* (3,556)	0.55* (2,588)	0.04 (104)
Heptachlor epoxide	—	—	—	—	1.0	0.67* (2,583)	− 0.10 (95)
Dieldrin	—	—	—	—	—	1.0	− 0.14 (73)
Time since cancer diagnosis	—	—	—	—	—	—	1.0

a Correlations between survival time (years) and OC pesticides among breast and prostate cancer cases.

* *p* < 0.05.

**Table 3:** Relationships between selected characteristics and OC pesticides [no. (%)].

	Age (years)			Race				Education				Poverty/income ratio				Smoking				BMI				Marital status			Alcohol consumption		
Chemical (%)	< 65	≥65	*p-*Value	NHW	NHB	Others	*p-*Value	< High school	High school	> High school	*p-*Value	< 1.0	1.0–2.0	> 2.0	*p-*Value	Never	Former	Current	*p-*Value	< 25.0	25–29	≥30	*p-*Value	Yes	No	*p-*Value	Yes	No	*p-*Value
β-HCH																													
< LOD	852 (30.7)	25 (2.9)	< 0.001	478 (27.1)	242 (35.7)	157 (18.5)	< 0.001	175 (19.6)	223 (27.6)	476 (28.5)	< 0.001	154 (32.0)	214 (27.8)	446 (25.4)	< 0.001	494 (29.5)	133 (16.5)	249 (30.5)	< 0.001	337 (34.8)	279 (24.4)	220 (19.2)	< 0.0001	381 (21.9)	471 (33.9)	< 0.001	587 (27.6)	224 (22.7)	< 0.001
< 33	903 (35.4)	74 (8.4)		555 (33.4)	197 (28.4)	225 (24.9)		232 (24.4)	220 (30.0)	525 (34.6)		146 (28.1)	185 (25.6)	571 (34.7)		491 (31.4)	216 (27.4)	269 (35.2)		331 (29.6)	354 (33.4)	292 (31.1)		552 (32.9)	388 (29.3)		710 (34.4)	211 (24.4)	
33–67	702 (23.5)	324 (37.3)		527 (26.6)	151 (19.4)	348 (25.6)		360 (27.5)	232 (26.3)	434 (24.6)		136 (19.5)	247 (22.8)	545 (27.7)		437 (21.1)	371 (36.8)	218 (23.4)		300 (20.9)	399 (26.8)	325 (29.6)		644 (29.4)	347 (19.6)		694 (25.4)	270 (26.2)	
> 67	483 (10.4)	506 (51.4)		348 (12.9)	153 (16.5)	488 (31.0)		499 (28.5)	196 (16.1)	291 (12.3)		214 (20.4)	304 (23.8)	361 (12.2)		557 (18.0)	300 (19.3)	131 (10.9)		255 (14.6)	348 (15.4)	382 (20.1)		523 (15.8)	423 (17.2)		514 (12.6)	408 (26.7)	

*p,p* ′*-*DDE																													
< LOD or < 33	1,208 (48.1)	80 (10.7)	< 0.001	867 (48.7)	255 (36.3)	170 (22.0)	< 0.001	233 (27.9)	318 (42.6)	739 (48.1)	< 0.001	190 (42.2)	280 (40.3)	740 (45.0)		672 (43.6)	250 (33.7)	368 (49.4)	< 0.001	484 (45.6)	422 (42.3)	384 (39.4)	< 0.0001	633 (39.2)	612 (47.5)	0.003	905 (45.9)	209 (34.6)	< 0.001
33–67	1,028 (35.8)	299 (40.1)		683 (36.5)	251 (34.8)	393 (33.4)		404 (35.8)	314 (37.7)	607 (36.1)		202 (32.2)	313 (34.2)	694 (30.0)		640 (35.1)	380 (40.3)	307 (35.2)		436 (35.3)	492 (37.5)	399 (36.7)		745 (39.3)	526 (32.2)		886 (358)	361 (36.3)	
> 67	735 (16.0)	555 (48.7)		381 (14.8)	245 (28.9)	664 (40.6)		643 (36.3)	247 (19.7)	398 (15.8)		260 (25.6)	361 (25.5)	520 (17.0)		679 (21.2)	405 (26.0)	205 (15.4)		365 (19.1)	470 (20.2)	450 (23.9)		745 (21.5)	508 (20.3)		743 (17.3)	454 (29.1	

Oxychlordane																													
< LOD	585 (19.0)	16 (1.3)	< 0.001	229 (13.8)	112 (18.0)	260 (25.7)	< 0.001	193 (17.1)	136 (14.9)	270 (16.7 )	< 0.001	134 (24.7)	167 (19.8)	256 (13.8)	< 0.001	378 (20.7)	88 (9.3)	134 (14.7)	< 0.001	247 (20.4)	193 (15.8)	160 (12.3)	0.001	291 (13.3)	305 (20.5)	< 0.001	356 (15.0)	192 (19.0)	< 0.001
< 33	959 (36.6)	36 (3.8)		479 (30.9)	192 (30.4)	324 (35.5)		248 (25.0)	245 (32.4)	501 (33.7)		188 (34.9)	201 (25.2)	529 (33.6)		526 (31.4)	213 (28.5)	255 (35.4)		341 (32.2)	353 (30.8)	300 (30.9)		546 (31.0)	443 (32.7)		688 (35.9)	233 (24.6)	
33–67	804 (32.0)	221 (26.8)		543 (33.7)	183 (27.9)	299 (23.3)		302 (25.3)	242 (32.3)	480 (33.0)		151 (22.7)	242 (27.8)	546 (34.1)		468 (29.4)	302 (32.8)	255 (33.4)		300 (28.8)	371 (32.2)	304 (33.1)		617 (33.9)	398 (27.6)		712 (32.7)	258 (28.5)	
> 67	397 (12.4)	605 (68.1)		514 (21.6)	198 (23.7)	290 (15.5)		451 (32.6)	209 (20.4)	340 (16.6)		159 (17.7)	292 (27.2)	448 (18.5)		477 (18.5)	355 (29.4)	169 (16.4)		281 (17.7)	376 (21.2)	341 (23.7)		547 (21.8)	438 (19.2)		587 (18.4)	360 (27.9)	

*trans*-Nonachlor																													
< LOD	318 (9.5)	3 (0.2)	< 0.001	128 (6.9)	50 (7.4)	143 (13.8)	< 0.001	102 (7.8)	70 (8.4)	149 (8.2)	< 0.001	72 (12.9)	82 (8.5)	142 (7.0)	< 0.001	205 (10.1)	37 (4.2)	78 (3.1)	< 0.001	134 (10.0)	103 (7.5)	83 (6.6)	< 0.001	135 (5.6)	176 (12.1)	< 0.001	197 (7.7)	103 (9.6)	< 0.001
< 33	1,144 (39.5)	31 (3.6)		573 (33.5)	213 (30.6)	387 (38.6)		291 (26.8)	281 (32.9)	601 (37.3)		209 (37.3)	26.6 (31.6)	614 (35.3)		665 (36.6)	221 (26.8)	288 (36.5)		433 (37.1)	309 (32.7)	352 (32.1)		643 (34.2)	429 (34.8)		787 (35.9)	303 (28.1)	
33–67	958 (35.3)	252 (30.6)		664 (37.1)	218 (32.0)	328 (26.0)		342 (30.0)	287 (34.8)	580 (36.2)		176 (28.1)	275 (31.2)	659 (37.1)		554 (32.5)	358 (37.5)	298 (35.9)		360 (31.4)	431 (34.9)	418 (38.1)		697 (36.6)	458 (30.6)		827 (35.8)	304 (31.8)	
> 67	534 (15.7)	646 (65.6)		557 (22.5)	264 (30.0)	359 (21.6)		531 (35.4)	245 (23.9)	401 (18.3)		195 (21.6)	326 (28.7)	522 (20.6)		561 (20.8)	408 (31.5)	210 (19.5)		349 (21.4)	455 (24.8)	372 (23.2)		630 (23.6)	507 (22.5)		709 (20.6)	407 (30.5)	

Heptachlor epoxide																													
< LOD	129 (47.3)	157 (19.2)	< 0.001	687 (42.0)	319 (49.5)	447 (44.2)	0.04	410 (40.2)	353 (45.9)	683 (43.0)	< 0.001	257 (47.3)	355 (45.4)	729 (42.0)	0.007	791 (46.1)	292 (33.8)	368 (46.9)	< 0.001	666 (60.6)	400 (39.9)	304 (26.8)	< 0.001	732 (38.7)	711 (49.2)	0.002	944 (43.1)	399 (42.2)	< 0.001
< 33	553 (21.6)	140 (15.7)		352 (20.8)	123 (19.2)	218 (21.0)		197 (17.5)	161 (19.5)	334 (22.4)		110 (17.6)	143 (16.1)	379 (23.0)		337 (19.0)	195 (23.6)	161 (21.2)		203 (16.9)	273 (24.1)	216 (26.8)		406 (21.9)	286 (19.3)		480 (22.2)	168 (16.0)	
33–67	507 (18.6)	232 (25.9)		364 (20.2)	112 (15.3)	259 (20.2)		252 (19.1)	156 (18.5)	320 (20.4)		120 (17.0)	184 (17.7)	368 (21.0)		339 (18.2)	238 (23.2)	158 (18.6)		155 (11.7)	288 (21.2)	290 (26.9)		447 (22.2)	282 (16.2)		507 (20.5)	184 (18.4)	
> 67	370 (12.5)	338 (39.2)		356 (17.0)	126 (16.0)	226 (14.6)		316 (23.2)	152 (16.1)	240 (14.2)		133 (18.1)	208 (20.8)	296 (14.0)		359 (16.6)	227 (19.4)	121 (13.3)		142 (10.8)	236 (14.8)	329 (25.0)		395 (17.2)	295 (15.3)		402 (14.2)	272 (23.4)	

Dieldrin																													
< LOD	567 (26.0)	44 (6.4)	< 0.001	268 (21.5)	130 (28.4)	213 (27.3)	0.02	177 (23.5)	147 (25.2)	286 (22.3)	0.06	124 (29.2)	153 (24.7)	301 (21.9)	0.09	325 (23.6)	122 (19.6)	164 (26.1)	0.004	303 (31.4)	203 (21.0)	103 (10.5)	< 0.001	317 (20.8)	294 (26.6)	0.20	400 (23.6)	169 (23.1)	0.01
< 33	546 (28.0)	110 (17.6)		361 (27.6)	111 (21.8)	184 (25.3)		179 (22.9)	155 (27.3)	320 (27.4)		115 (30.5)	158 (27.3)	338 (25.7)		331 (25.8)	151 (24.3)	173 (29.8)		243 (29.1)	245 (28.2)	166 (21.8)		361 (27.4)	295 (25.4)		429 (26.4)	186 (27.2	
33–67	518 (27.6)	161 (26.6)		349 (27.3)	139 (26.7)	191 (28.4)		205 (25.5)	141 (25.8)	332 (28.8)		92 (20.7)	158 (26.2)	382 (29.6)		324 (28.2)	194 (27.9)	161 (25.7)		182 (22.0)	232 (26.3)	264 (34.9)		388 (27.8)	291 (27.0)		473 (28.4)	159 (23.3)	
> 67	373 (18.4)	291 (49.4)		368 (23.6)	128 (23.1)	174 (19.0)		242 (28.1)	150 (21.7)	270 (21.5)		97 (19.6)	167 (21.8)	353 (22.8)		329 (22.4)	215 (28.2)	125 (18.4)		135 (12.5)	241 (24.5)	293 (32.8)		385 (24.0)	285 (21.0)		420 (21.6)	210 (26.4)	

Abbreviations: NHB, non-Hispanic black; NHW, non-Hispanic white.

**Table 4:** Distribution of selected characteristics by cancer status among the 1999–2004 NHANES adult participants ≥20 years of age [no. (%)].

	Breast cancer			Prostate cancer		
Characteristic	Cases	Noncases	*p-*Value	Cases	Noncases	*p-*Value
Age (years)						
< 65	21 (42.5)	1,701 (84.4)	< 0.0001	7 (14.3)	1,492 (88.3)	< 0.001
≥65	41 (57.5)	488 (15.6)		58 (85.7)	428 (11.7)	

Race						
Non-Hispanic white	52 (92.6)	1,033 (69.1)	< 0.001	46 (83.2)	948 (72.1)	0.02
Non-Hispanic black	7 (6.7)	437 (12.4)		13	372 (10.5)	
Others	3 (0.7)	719 (18.5)		6 (6.1)	600 (17.4)	

Education						
< High school	17 (21.8)	693 (20.0)	0.19	26 (30.2)	650 (20.6)	< 0.001
High School	19 (35.7)	502 (24.8)		13 (22.9)	426 (24.6)	
> High school	26 (42.5)	991 (55.1)		26 (46.8)	841 (54.8)	

Poverty/income ratio						
< 1.0	5 (5.5)	410 (16.2)	0.02	3 (2.9)	300 (11.4)	< 0.001
1.0–2.0	17 (26.5)	538 (22.8)		16 (28.4)	460 (20.1)	
> 2.0	36 (68.0)	1,037 (61.0)		39 (68.7)	989 (68.4)	

Smoking						
Never	31 (47.8)	1,327 (56.7)	0.20	26 (39.1)	797 (42.7)	0.006
Former	25 (38.3)	474 (21.8)		31 (50.1)	585 (27.3)	
Current	6 (13.9)	387 (21.5)		7 (10.8)	537 (30.0)	

BMI						
< 25.0	24 (39.8)	747 (37.9)	0.51	20 (25.5)	605 (31.7)	0.41
25–29	22 (28.7)	667 (36.7)		30 (48.8)	778 (38.9)	
≥30	16 (31.5)	769 (25.4)		15 (25.7)	535 (29.4)	

Marital status						
Yes	23 (43.6)	1,107 (55.2)	0.21	44 (70.0)	1,116 (61.0)	0.11
No	38 (56.4)	1,001 (44.8)		20 (30.0)	744 (39.0)	

Alcohol consumption						
Yes	39 (68.3)	1,123 (61.2)	0.39	45 (68.4)	1,499 (83.6)	0.04
No	22 (31.7)	876 (38.8)		17 (31.6)	311 (16.4)	

**Table 5:** Lipid-adjusted serum concentration (ng/g) and prevalent prostate cancer in 1999–2004 NHANES adult participants, by tertile.

	Not detectable		Detectable		
Chemical	< LOD	< 33rd	33rd –67th	> 67th	*p*_trend_
β-HCH					
Median concentration	—	6.2	16.2	53.9	
Cases/no.	5/453	9/528	23/531	21/307	
Age-adjusted OR (95% CI)	1.0	1.0	1.44 (0.55–3.78)	3.10 (1.36–7.11)	0.007
Adjusted OR (95% CI)^a^	1.0	1.0	1.46 (0.52–4.13)	3.36 (1.24–9.10)	0.02

*p,p* ′*-*DDE					
Median concentration	—	113.0	386.0	1530.0	
Cases/no.	0/3	8/599	28/692	23/547	
Age-adjusted OR (95% CI)	1.0	1.0	1.89 (0.71–5.04)	2.02 (0.76–5.32)	0.16
Adjusted OR (95% CI)^a^	1.0	1.0	2.05 (0.76–5.50)	2.64 (0.92–7.57)	0.07

Oxychlordane					
Median concentration	—	8.0	17.2	37.0	
Cases/no.	0/273	5/488	19/506	29/441	
Age-adjusted OR (95% CI)	1.0	1.0	3.38 (1.02–11.3)	3.39 (1.03–11.1)	0.04
Adjusted OR (95% CI)^a^	1.0	1.0	3.54 (0.91–13.8)	3.54 (0.92–13.6)	0.06

*Trans*-nonachlor					
Median concentration	—	9.9	24.8	56.4	
Cases/no.	0/154	3/521	12/571	41/582	
Age-adjusted OR (95% CI)	1.0	1.0	4.76 (1.13–20.1)	9.51 (2.22–40.8)	0.002
Adjusted OR (95% CI^a^	1.0	1.0	5.84 (1.06–32.2)	14.1 (2.55–77.9)	0.002

Heptachlor epoxide					
Median concentration	—	5.0	8.9	18.0	
Cases/no.	10/681	7/339	12/360	21/311	
Age-adjusted OR (95% CI)	1.0	0.68 (0.20–2.29)	1.39 (0.57–3.34)	1.79 (0.59–5.47)	0.15
Adjusted OR (95% CI)^a^	1.0	1.01 (0.31–3.20)	1.65 (0.73–3.76)	1.91 (0.70–5.33)	0.12

Dieldrin					
Median concentration	—	4.8	7.9	14.7	
Cases/no.	0/257	6/286	6/362	25/343	
Age-adjusted OR (95% CI)	1.0	1.0	0.98 (0.31–3.08)	2.69 (1.09–6.68)	0.03
Adjusted OR (95% CI)^a^	1.0	1.0	1.06 (0.30–3.73)	2.74 (1.01–7.49)	0.04

a Adjusted for age, race, and ethnicity, BMI, education, smoking, data cycle, and marital status.

**Table 6:** Lipid-adjusted serum concentration (ng/g) and prevalent breast cancer in 1999–2004 NHANES adult participants, by tertile.

	Not detectable		Detectable		
Chemical	< LOD	< 33rd	33rd –67th	> 67th	*p*_trend_
β-HCH					
Median concentration	—	6.2	16.2	53.9	
Cases/no.	2/424	5/449	23/495	25/682	
Age-adjusted OR (95% CI)	1.0	1.0	2.84 (0.79–10.2)	1.67 (0.42–6.54)	0.46
Adjusted OR (95% CI)^a^	1.0	1.0	2.82 (0.75–10.65)	2.33 (0.53–10.33)	0.26

*p,p* ′*-*DDE					
Median concentration	—	113.0	386.0	1530.0	
Cases/no.	0/1	9/689	20/635	26/743	
Age-adjusted OR (95% CI)	1.0	1.0	1.24 (0.48–3.19)	0.93 (0.34–2.56)	0.88
Adjusted OR (95% CI)^a^	1.0	1.0	1.26 (0.49–3.27)	1.19 (0.40–3.50)	0.76

Oxychlordane					
Median concentration	—	8.0	17.2	37.0	
Cases/no.	2/328	3/507	18/519	28/561	
Age-adjusted OR (95% CI)	1.0	1.0	4.25 (1.25–14.4)	2.97 (0.88–10.0)	0.08
Adjusted OR (95% CI)^a^	1.0	1.0	3.68 (1.12–12.13)	2.55 (0.73–8.83)	0.14

*Trans*-nonachlor					
Median concentration	—	9.9	24.8	56.4	
Cases/no.	1/167	4/654	21/639	29/598	
Age-adjusted OR (95% CI)	1.0	1.0	3.29 (0.96–11.3)	2.66 (0.63–11.2)	0.18
Adjusted OR (95% CI)^a^	1.0	1.0	2.80 (0.81–9.67)	2.60 (0.65–10.35)	

Heptachlor epoxide					
Median concentration	—	5.0	8.9	18.0	
Cases/no.	12/772	16/354	11/375	13/397	
Age-adjusted OR (95% CI)	1.0	2.75 (1.26–5.99)	1.14 (0.46–2.84)	1.19 (0.50–2.83)	0.79
Adjusted OR (95% CI)^a^	1.0	2.21 (1.01–4.83)	1.05 (0.36–3.05)	1.08 (0.40–2.93)	0.74

Dieldrin					
Median concentration	—	4.8	7.9	14.7	
Cases/no.	5/354	16/370	12/317	10/327	
Age-adjusted OR (95% CI)	1.0	2.18 (0.83–5.76)	1.23 (0.38–4.02)	1.01 (0.30–3.41)	0.78
Adjusted OR (95% CI)^a^	1.0	2.28 (0.76–6.87)	1.21 (0.34–4.29)	1.04 (0.26–4.16)	0.81

a Adjusted for age, race, and ethnicity, BMI, education, smoking, data cycle, and marital status.
